# Effects of Tai Chi on pain, functional dysfunction, and sleep in patients with chronic nonspecific low back pain: a systematic review and meta-analysis

**DOI:** 10.3389/fpain.2026.1852975

**Published:** 2026-07-02

**Authors:** Cenhui Xu, Huaimeng Cui, Tongtong Hao

**Affiliations:** 1 School of Physical Education, Jiangsu Normal University, Xuzhou, China; 2 College of Chinese Wushu, Beijing Sport University, Beijing, China

**Keywords:** chronic nonspecific low back pain, functional dysfunction, meta-analysis, pain, sleep quality, Tai Chi

## Abstract

**Objective:**

This study systematically evaluated the effects of Tai Chi on pain, functional disability, and sleep quality in patients with chronic non-specific low back pain (CNLBP).

**Methods:**

We searched CNKI, WanFang, VIP, China Biology Medicine Database, PubMed, Web of Science, Embase, and the Cochrane Library for randomized controlled trials (RCTs) of Tai Chi for CNLBP. Study quality was assessed using the Cochrane RoB 2 tool and the PEDro scale. Meta-analysis was used to pool the data.

**Results:**

Eight RCTs involving 526 patients with CNLBP were included. Meta-analysis showed that Tai Chi significantly reduced pain, improving VAS scores (MD = −1.40, 95% CI: −2.41 to −0.40) and NRS scores. Tai Chi also significantly improved RMDQ scores (MD = −1.67, 95% CI: −2.75 to −0.59) and overall functional disability (SMD = −0.51, 95% CI: −0.90 to −0.12), but did not significantly affect ODI scores (MD = −0.62, 95% CI: −2.53 to 1.29). Moreover, improvements in PSQI scores were not statistically significant (MD = −0.18, 95% CI: −1.82 to 1.45).

**Conclusion:**

Tai Chi can effectively relieve pain and improve functional disability in patients with CNLBP. It is a safe and feasible non-pharmacological rehabilitation approach. However, current evidence is insufficient to confirm its effect on sleep quality. Further high-quality RCTs are needed.

**Systematic Review Registration:**

PROSPERO CRD420261361228.

## 1 Introduction

Chronic nonspecific low back pain (CNLBP) refers to low back pain lasting more than 12 weeks without a clear, identifiable specific pathological cause (such as infection, tumor, fracture, or nerve root syndrome) (1). It is a leading cause of functional disability and loss of productivity worldwide. Epidemiological surveys indicate that the lifetime prevalence of CNLBP is as high as 60%–80%. With population aging and lifestyle changes, its incidence continues to rise (2). CNLBP not only causes persistent pain but also significantly limits trunk mobility, leading to functional dysfunction. It is often accompanied by comorbidities such as sleep disorders, imposing a heavy burden on individuals, families, and healthcare systems (3).

Currently, clinical management of CNLBP primarily relies on medication (e.g., non-steroidal anti-inflammatory drugs, muscle relaxants), physical therapy (e.g., core stability training, manual therapy), and cognitive-behavioral interventions (4). However, long-term medication use carries gastrointestinal, cardiovascular, and dependency risks, while conventional rehabilitation often faces limited compliance. Some patients experience insignificant symptom improvement or frequent recurrences (5). Therefore, seeking safe, effective, and sustainable non-pharmacological treatments has become a key direction in CNLBP research.

Tai Chi, which integrates dynamic meditation, breathing techniques, and relaxation, has become an important tool for the clinical rehabilitation of various chronic pain conditions (6, 7). Although preliminary evidence supports the benefit of Tai Chi for clinical outcomes in CNLBP patients, existing research still faces multiple limitations: first, there is high heterogeneity in intervention parameters and control designs; second, systematic reviews targeting sleep disorders—a core comorbidity—are currently lacking (8). Given the contradictions in individual study results, a comprehensive quantitative analysis based on high-level evidence is essential to clarify the clinical rehabilitative value of Tai Chi.

Therefore, this systematic review and meta-analysis aims to systematically integrate evidence from existing randomized controlled trials (RCTs) and quantitatively evaluate the intervention effects of Tai Chi on CNLBP patients across three key outcome measures: pain intensity, functional dysfunction, and sleep quality. This study aims to provide an evidence-based foundation for clinical practice and the development of future rehabilitation guidelines.

## 2 Methods

### 2.1 Registration and procedural protocol

This meta-analysis was designed and implemented in accordance with the Preferred Reporting Items for Systematic Reviews and Meta-Analyses (PRISMA) guidelines (9). The study protocol has been registered in the International Prospective Register of Systematic Reviews (PROSPERO) under registration number CRD420261361228.

### 2.2 Search strategy

Databases including CNKI, VIP, Wanfang, CBM, PubMed, Cochrane Library, Embase, and Web of Science were searched from their inception to March 2026, with the final search conducted on March 29, 2026. All databases were searched using the same PICOS framework and core search terms. The search strategy combined both subject headings and free-text terms. Key terms included: “chronic non-specific low back pain,” “chronic low back pain,” “non-specific low back pain,” “low back pain,” and “lumbago,” as well as “Tai Chi,” “Tai Ji Quan,” “Tai Chi Chuan,” “Tai Ji,” “Yang-style Tai Chi,” and “Qigong.” The same PICOS framework and core search terms were applied across all databases. The search strategy combined subject headings with free-text terms and was appropriately adapted to the syntax requirements of each database to ensure consistency and reproducibility of the retrieval process. In addition, to minimize potential publication bias, gray literature sources, including dissertations and theses, were also searched. Manual reference screening was further conducted to identify potentially eligible studies. The detailed search strategy for PubMed is presented in Table 1, while the search strategies for the other databases are available upon request.

**Table 1:** Search strategy for pubMed database.

Number	Search terms
#1	chronic low back pain
#2	low back pain
#3	non-specific low back pain
#4	OR/#1-#3
#5	Tai Chi
#6	Tai Ji Quan
#7	Qigong
#8	OR/#5-#7
#9	pain
#10	disability
#11	sleep
#12	OR/#9-#11
#13	randomized controlled trial
#14	RCT
#15	OR/#13-#14
#16	#4 AND #8 AND #12 AND #15

### 2.3 Inclusion and exclusion criteria

To ensure a rigorous selection process, inclusion and exclusion criteria were established utilizing the PICOS strategy (10). Detailed descriptions of these criteria, alongside the primary and secondary outcome indicators, are detailed in Table 2.

**Table 2:** Inclusion and exclusion criteria.

Parameter	Inclusion Criteria	Exclusion Criteria
P (Participants)	Adults (≥18 years old) with a clinical diagnosis of chronic nonspecific low back pain (course of disease ≥12 weeks); no restrictions on gender, ethnicity, or duration of the condition.	Patients with specific low back pain (e.g., lumbar disc herniation, spondylolisthesis, spinal stenosis, fractures, tumors, infections, ankylosing spondylitis); individuals with severe comorbidities of the heart, liver, or kidneys; pregnant or lactating women.
I (Intervention)	Tai Chi: including various styles (e.g., Yang, Chen, Wu, Sun styles); must include detailed records of practice frequency, duration per session, and total duration.	We excluded literature that did not involve physical exercise, lacked specific dosage data (e.g., frequency or duration), or integrated Tai Chi with other treatments in a manner that confounded the isolation of its unique impact.Studies involving combined interventions were included only when the co-interventions were equally applied in both intervention and control groups, allowing the additional effect of Tai Chi to be relatively isolated.
C (Comparison)	Control groups receiving routine rehabilitation, conventional care, health education, waiting list, no intervention, or other non-Tai Chi exercises (e.g., core stability training, walking, stretching).	Trials were excluded if they utilized a single-group design without a control comparison or combined Tai Chi with extensive ancillary treatments that obscured the isolation of the specific exercise dosage.
O (Outcomes)	Core Indicators: Pain intensity (e.g., VAS, NRS scores), functional dysfunction (e.g., RMDQ, ODI scores), and sleep quality (e.g., PSQI scores).	Literature from which raw data cannot be extracted or where required outcome measures are not reported.
S (Setting/Study Design)	The scope of this review was limited to publicly available randomized controlled trials, with eligibility restricted to articles authored in the English or Chinese languages.	We excluded reviews, case series, conference proceedings, non-peer-reviewed reports, and overlapping or duplicate records.

### 2.4 Literature screening and data extraction

Literature screening and data extraction were performed independently by two researchers (Cenhui Xu and Tongtong Hao), with results cross-verified. Any discrepancies were resolved through discussion or by consulting a third researcher (Huaimeng Cui) (11). Initial screening was conducted by title to exclude irrelevant studies, followed by a detailed review of abstracts and full texts to determine eligibility (12). When necessary, authors of the original studies were contacted via email or telephone to clarify ambiguous information.

The following data were extracted:

1. Basic study information: Title, first author, and year of publication.
2. Participant characteristics: Sample size per group, age, gender, and duration of disease.
3. Intervention details: Tai Chi style, intervention period, frequency, duration per session, and control measures.
4. Risk of bias assessment elements.
5. Outcome measures and results: Mean, standard deviation, and sample size for pain intensity, functional dysfunction, and sleep quality (9).

For studies reporting multiple follow-up time points, post-intervention outcome data were preferentially extracted for Meta-analysis to improve comparability across studies. If post-intervention data were not explicitly reported, the time point closest to the end of the intervention was selected for analysis.

### 2.5 Risk of bias assessment

Two researchers (Cenhui Xu and Tongtong Hao) independently evaluated the methodological quality of the included studies and cross-verified the results. Any discrepancies were resolved through consultation with a third researcher (Huaimeng Cui). The Cochrane Risk of Bias tool version 2 (RoB 2) (13), second edition, was utilized to assess the risk of bias across five domains: bias arising from the randomization process, bias due to deviations from intended interventions, bias due to missing outcome data, bias in measurement of the outcome, and bias in selection of the reported result (9). In addition, the Physiotherapy Evidence Database (PEDro) scale was employed to evaluate the risk of bias and methodological quality of the included studies, with scores ranging from 0 to 10 (14). According to the evaluation criteria, one point is awarded for each item met between items 2 and 11 (maximum score of 10). Studies with a score of ≥6 are categorized as high quality, scores of 4–5 as moderate quality, and scores of ≤3 as low quality.

### 2.6 Statistical analysis

Data synthesis was executed via SPSSAU software (15). To evaluate continuous outcomes, Mean Difference (MD) or Standardized Mean Difference (SMD) was calculated with corresponding 95% Confidence Intervals (CIs) (16). The choice between MD and SMD depended on whether the assessment tools across studies were uniform (17). The *I*^2^ statistic served to quantify inter-study heterogeneity. A fixed-effects model was utilized when heterogeneity was considered acceptable (*I*^2^ < 50% and *P* > 0.1). Conversely, if significant variability was detected (*I*^2^ ≥ 50% or *P* ≤ 0.1), a random-effects model was implemented for pooling (18). The stability of the synthesized results was verified through sensitivity analysis, conducted by systematically omitting one study at a time and re-estimating the effect size (19). Due to the limited number of included studies (<10), formal assessment of publication bias was not performed, as statistical tests may be unreliable. Statistical significance was defined as *P* < 0.05 across all analyses (20).

## 3 Results

### 3.1 Literature selection process

**Figure 1:**
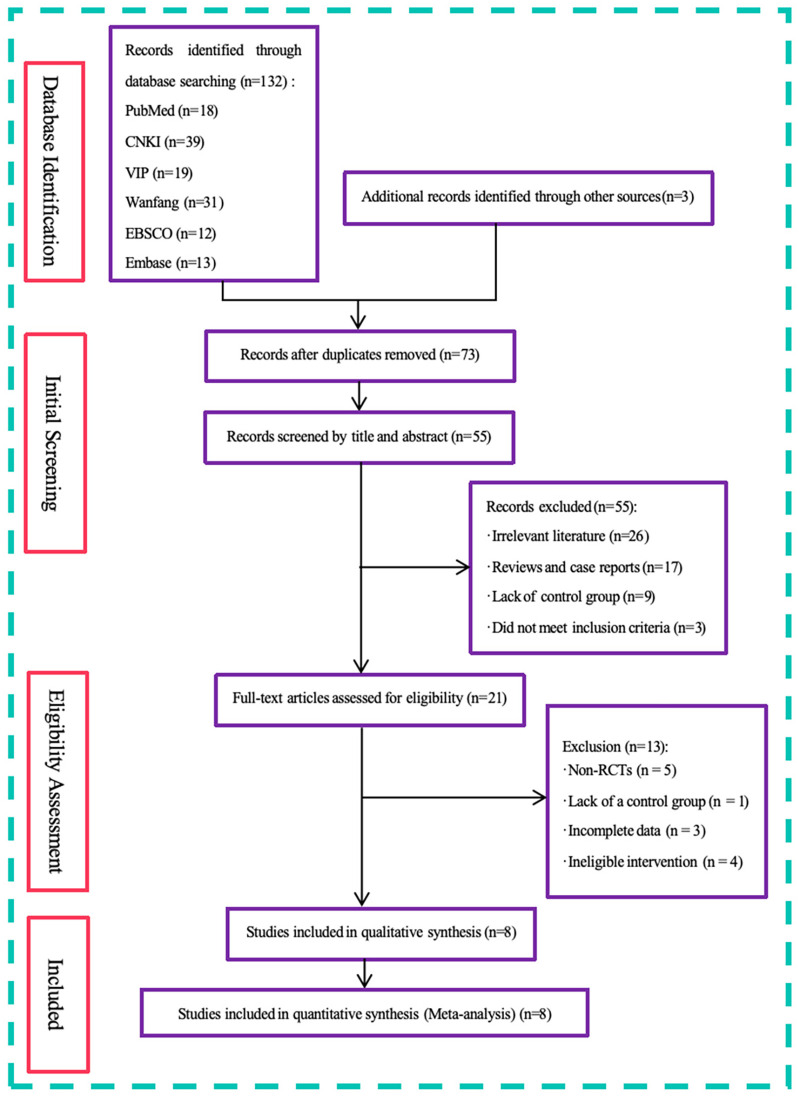
Literature screening process and results.

The initial search across various databases yielded 132 records, with an additional 3 studies identified through manual reference tracing. After merging and removing duplicates using EndNote X9, 73 unique records remained. Preliminary screening of titles and abstracts resulted in the exclusion of 55 irrelevant studies, leaving 21 articles for full-text evaluation. Upon detailed review, 13 studies were excluded for the following reasons: non-RCT design (*n* = 5), absence of a control group (*n* = 1), incomplete data (*n* = 3), and inappropriate intervention (*n* = 4). Ultimately, 8 studies met all criteria and were included in this meta-analysis. The systematic selection process and corresponding results are illustrated in the PRISMA flow diagram (Figure 1).

### 3.2 Characteristics of included studies

A total of eight RCTs involving 526 patients with CNLBP were ultimately included. The studies were published between 2011 and 2024, and the mean age of participants ranged from 21 to 61 years. The Tai Chi interventions included several styles, such as the 24-form Simplified Tai Chi, Yang-style Tai Chi, Chen-style Tai Chi, and modified Tai Chi. The intervention duration ranged from 4 to 12 weeks, with training frequencies of 2–5 sessions per week and session lengths of 30–65 min. Control interventions included health education, usual care, sham Tai Chi, core stability training, acupuncture, and no-exercise interventions. Pain, functional disability, and sleep quality were primarily assessed using the Visual Analog Scale (VAS), Numeric Rating Scale (NRS), Roland–Morris Disability Questionnaire (RMDQ), Oswestry Disability Index (ODI), and Pittsburgh Sleep Quality Index (PSQI). Detailed study characteristics are presented in Table 3.

**Table 3:** Baseline profiles and intervention protocols of the selected trials.

First author	Year	Mean age (I/C, years)		Sample size (I/C, n)		Intervention description (I/C)		Intervention dosage	Outcome measures	Follow-up
		Intervention group	Control group	Intervention group	Control group	Intervention group	Control group			
Chang Xiaolong (21)	2024	39.796 ± 12.233	36.444 ± 10.629	54	54	Tai Chi for Strengthening the Waist (Tai Ji Qiang Yao Fa)	Health education, guidance, and Q&A.	12W-3/week-60 min	Pain (NRS), functional dysfunction (RMDQ), sleep quality (PSQI)	Week 12 follow-up
Wang Rui (22)	2021	23.80 ± 2.67	23.47 ± 2.84	10	10	Simplified Tai Chi	Sham Tai Chi (5 min) followed by 35 min of rest. Sham Tai Chi involved simple physical movements without mental focus or breathing coordination.	12W-3/week-60 min	Pain (NRS), functional dysfunction (RMDQ), sleep quality (PSQI)	1 h after the first treatment; follow-up duration: 30–45 min
Wang Rui (23)	2024	21.86 ± 2.85	23.40 ± 4.10	37	36	Simplified Tai Chi	No exercise intervention.	12W-3/week-60 min	Pain (NRS), functional dysfunction (ODI), sleep quality (PSQI)	Week 36 follow-up
Tong Xiao (24)	2017	42.3 ± 4.26	41.6 ± 4.09	35	36	Modified Tai Chi “Shan Tong Bei” method	Conventional core muscle exercises.	12W-3/week-30 min	Pain (VAS)	3-month follow-up
Li Mengke (25)	2024	21.08 ± 1.26	20.75 ± 1.14	13	12	24-form Simplified Tai Chi	Education on low back pain; no intervention; maintenance of daily activities.	8W-3/week-45 min	Pain (VAS), functional dysfunction (RMDQ)	None
Wu Tingting (26)	2017	39.08 ± 12.56	36.81 ± 9.56	25	16	Yang-style 24-form Tai Chi combined with acupuncture therapy.	Acupuncture therapy only.	4W-5/week-65 min	Pain (VAS), functional dysfunction (ODI)	3-month follow-up
Liu Jing (5)	2019	58.13 ± 5.38	60.67 ± 2.58	15	13	Chen-style 16-form Tai Chi	No rehabilitation intervention; maintenance of original lifestyle.	12W-3/week-60 min	Pain (VAS)	None
Hall AM (27)	2011	43.4 ± 13.5	44.3 ± 13.0	80	80	A health-oriented Tai Chi program adapted by Paul Lam.	Usual Care	10W-2/week-40 min	Pain (VAS), functional dysfunction (RMDQ)	10 weeks

I, intervention group; C, control group; W, weeks; min, minutes. VAS/NRS, visual analogue/numeric rating scale; ODI/RMDQ, oswestry disability index/roland-morris disability questionnaire; PSQI, pittsburgh sleep quality index; SF-12/36, short form-12/36 health survey. Data are presented as mean ± SD or *n*.

### 3.3 Risk of bias and quality assessment

The Cochrane Risk of Bias 2 tool (RoB 2) was used to assess the overall risk of bias of the 8 included studies. According to the Cochrane Risk of Bias 2 (RoB 2) assessment, most included studies were rated as having either a low risk of bias or some concerns, primarily due to insufficient information regarding allocation concealment, participant blinding, or incomplete reporting of outcome data. Given the nature of exercise-based interventions, blinding of participants and personnel was difficult to implement in several trials. Nevertheless, no study was judged to have a high overall risk of bias. Overall, the methodological quality of the included studies was considered acceptable and suitable for meta-analysis.

**Figure 2:**
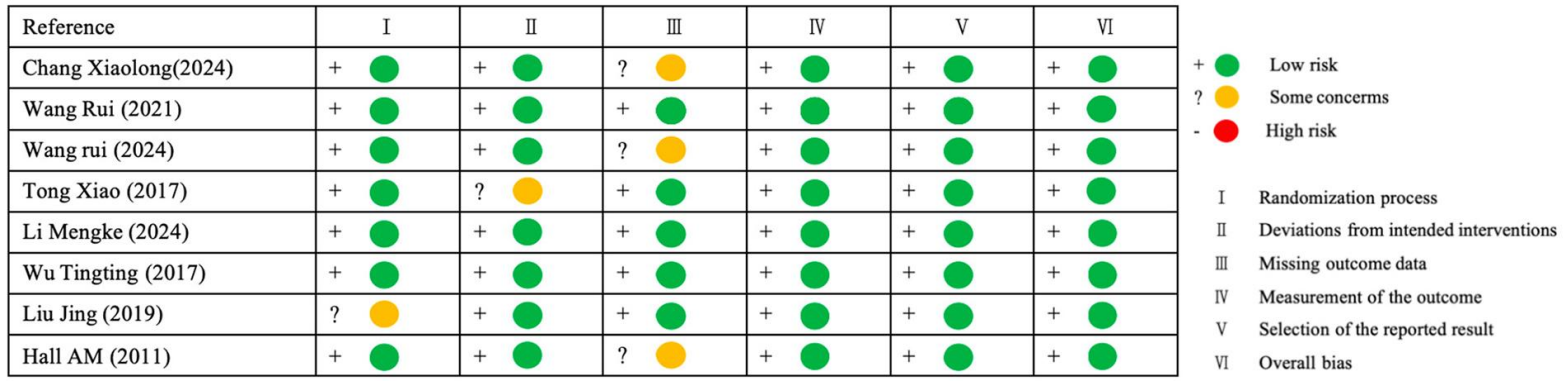
Quality assessment of the included studies based on the RoB 2 tool.

According to the PEDro scale, the included studies scored between 6 and 8 points, with a mean score of 6.75. Three studies—Wang Rui (22), Wu Tingting (26), and Liu Jing (5)—received a score of 8, while the remaining five studies scored 6 points. Based on the PEDro quality classification criteria, all included studies were considered high-quality studies. Overall, the methodological quality of the included literature was relatively high, indicating good overall study quality (Table 4 and Figure 2).

**Table 4:** PEDro methodological quality assessment of the included studies.

Study	PEDro scale											
	①	②	③	④	⑤	⑥	⑦	⑧	⑨	⑩	⑪	score
Chang Xiaolong (21)	Yes	1		1				1	1	1	1	6
Wang Rui (22)	Yes	1		1	1		1	1	1	1	1	8
Wang Rui (23)	Yes	1		1				1	1	1	1	6
Tong Xiao (24)	Yes	1		1				1	1	1	1	6
Li Mengke (25)	Yes	1		1				1	1	1	1	6
Wu Tingting (26)	Yes	1		1				1	1	1	1	8
Liu Jing (5)	Yes	1		1	1		1	1	1	1	1	8
Hall AM (27)	Yes	1		1				1	1	1	1	6

① Selection criteria were clearly stated; ② Subjects were randomized; ③ Hidden allocation process; ④ Initial similarity in key prognostic factors; ⑤ Masking of participants; ⑥ Masking of providers; ⑦ Masking of outcome evaluators; ⑧ Completion of follow-up by ≥85% of sample; ⑨ Analysis by original assigned groups [Intention-to-treat analysis (ITT)]; ⑩ Results of between-group tests; ⑪ Inclusion of effect size means and variability.

### 3.4 Effects of Tai Chi on pain intensity in patients with CNLBP

All eight included studies reported pain intensity outcomes. Five studies assessed pain intensity using the Visual Analog Scale (VAS), whereas three studies used the Numeric Rating Scale (NRS) to evaluate average pain and worst pain. The findings of the meta-analyses for VAS scores, NRS average pain scores, and NRS worst pain scores, together with the subgroup analyses of VAS scores, are presented in the following sections.

#### 3.4.1 Pain intensity: VAS score

**Figure 3:**
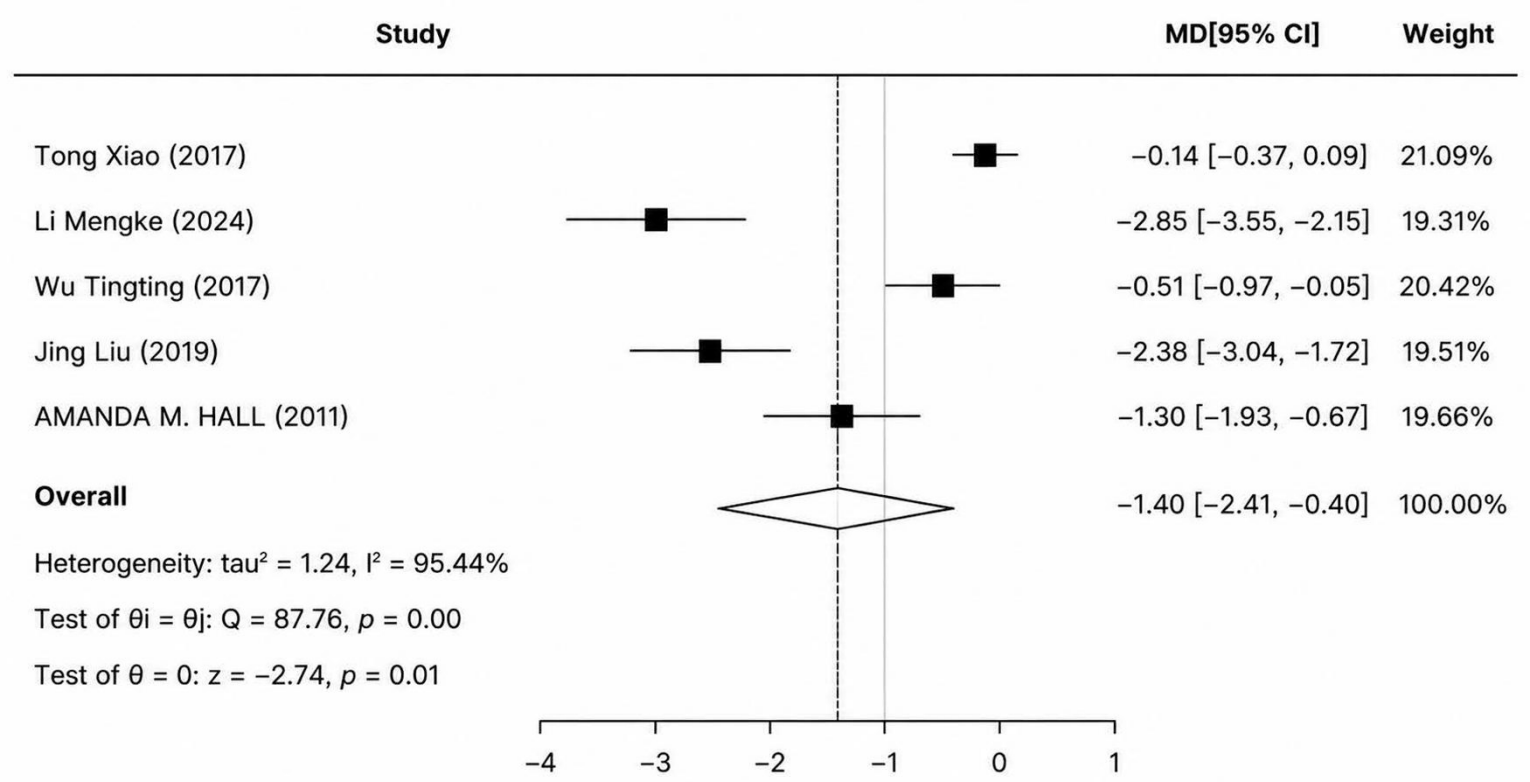
Forest plot illustrating the impact of Tai Chi on VAS pain intensity among patients with CNLB.

A total of five randomized controlled trials involving 325 patients with chronic non-specific low back pain (CNLBP) were included to evaluate the effects of Tai Chi on pain intensity measured by the Visual Analog Scale (VAS) (Figure 3). All studies reported reductions in VAS scores following Tai Chi intervention, with the largest decreases observed in the studies by Li Mengke (25) and Jing Liu (5). A random-effects model was used for the pooled analysis. The results showed a significant reduction in pain intensity in the Tai Chi group compared with the control group (MD = −1.40, 95% CI: −2.41 to −0.40; Z = 2.74, *p* = 0.01), indicating that Tai Chi effectively alleviates pain in patients with CNLBP. Substantial heterogeneity was observed among the included studies (*I*^2^ = 95.44%, H = 4.68, τ^2^ = 1.24). This heterogeneity may be related to differences in intervention duration, sample size, and baseline characteristics of participants. Sensitivity analysis demonstrated that the pooled effect remained relatively stable after sequential exclusion of individual studies, with MD values ranging from −1.04 to −1.74, suggesting that the findings were robust. Publication bias was not formally assessed due to the small number of studies, and thus these analyses are not reported.

Overall, the findings suggest that Tai Chi may reduce pain intensity in patients with CNLBP. However, the results should be interpreted with caution because of the substantial heterogeneity across studies and the potential presence of publication bias. Further large-scale, multicenter randomized controlled trials are warranted to confirm these findings.

Due to the high heterogeneity observed in the VAS meta-analysis (*I*^2^ = 95.44%), subgroup analyses were performed according to intervention duration, training frequency, session length, and intervention type to explore potential sources of heterogeneity (Table 5).

**Table 5:** Subgroup analysis of the effects of Tai Chi on VAS pain scores in patients with chronic non-specific low back pain.

Outcome	Moderators	Subgroup	No. of Studies (k)	Effect Size Hedges' g [95% CI]	I²
VAS Score	Duration (cov1)	Subgroup 1 (≤8 weeks)	2	−1.664 [−3.957, 0.629]	95.44%
		Subgroup 2 (>8 weeks)	3	−1.247 [−2.635, 0.140]	60.27%
	Frequency (cov2)	Subgroup 1 (≤3 times/week)	4	−1.646 [−3.046, −0.246]	79.58%
		Subgroup 2 (>3 times/week)	1	−0.510 [−0.967, −0.053]	0.00%
	Session Length (cov3)	Subgroup 1 (≤45 min)	3	−1.404 [−2.998, 0.190]	60.07%
		Subgroup 2 (>45 min)	2	−1.429 [−3.261, 0.403]	39.93%
	Intervention Type (cov4)	Subgroup 1 (Pure Tai Chi)	4	−1.646 [−3.046, −0.246]	79.58%
		Subgroup 2 (Combined Tai Chi)	1	−0.510 [−0.967, −0.053]	0.00%

Subgroup analyses were performed according to intervention duration (≤8 weeks vs. >8 weeks), training frequency (≤3 times/week vs. >3 times/week), session length (≤45 min vs. >45 min), and intervention type (pure Tai Chi vs. combined Tai Chi intervention). Negative Hedges' g values indicate greater reductions in pain intensity in the Tai Chi group compared with the control group.

For intervention duration, a larger effect size was observed in the ≤8-week subgroup (Hedges' *g* = −1.664, 95% CI: −3.957 to 0.629) than in the >8-week subgroup (Hedges' *g* = −1.247, 95% CI: −2.635 to 0.140). However, both confidence intervals crossed zero, indicating that the subgroup-specific effects were not statistically significant.

For training frequency, favorable effects were observed in both the ≤3 sessions/week subgroup (Hedges' *g* = −1.646, 95% CI: −3.046 to −0.246) and the >3 sessions/week subgroup (Hedges' *g* = −0.510, 95% CI: −0.967 to −0.053). However, the latter subgroup included only one study.

For session length, the effect estimates were similar between the ≤45 min subgroup (Hedges' *g* = −1.404, 95% CI: −2.998 to 0.190) and the >45 min subgroup (Hedges' *g* = −1.429, 95% CI: −3.261 to 0.403). Neither subgroup reached statistical significance.

Regarding intervention type, both pure Tai Chi (Hedges' *g* = −1.646, 95% CI: −3.046 to −0.246) and combined Tai Chi interventions (Hedges' *g* = −0.510, 95% CI: −0.967 to −0.053) were associated with reductions in pain intensity. However, the combined-intervention subgroup was represented by a single study.

Overall, the subgroup analyses suggested that intervention duration, training frequency, session length, and intervention type may contribute to variations in treatment effects. Nevertheless, the limited number of studies within several subgroups and the substantial heterogeneity observed warrant cautious interpretation of these findings.

#### 3.4.2 Pain intensity: average NRS score

**Figure 4:**
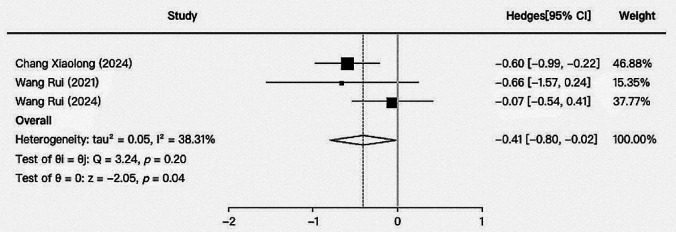
Forest plot of the effect of Tai Chi on average NRS pain scores in patients with chronic nonspecific low back pain.

Three studies (Chang Xiaolong (21), Wang Rui (22), and Wang Rui (23)) provided data for the average dimension of pain intensity measured by the Numeric Rating Scale (NRS). Heterogeneity testing revealed *I*^2^ = 38.31% and *P* = 0.20, indicating mild heterogeneity; therefore, a fixed-effects model was utilized for the meta-analysis. The results demonstrated that Tai Chi significantly reduced NRS scores compared to the control group (Hedges' *g* = −0.41, 95% CI: −0.80 to −0.02, *P* = 0.04). Subgroup analysis showed a positive trend for pain reduction in both Chang Xiaolong 2024 (Hedges' *g* = −0.60, 95% CI: −0.99 to −0.22) and Wang Rui 2021 (Hedges' *g* = −0.66, 95% CI: −1.57 to 0.24). Specifically, the effect in Chang Xiaolong 2024 reached statistical significance. In contrast, the effect size for Wang Rui 2024 was smaller and did not achieve statistical significance (Hedges' *g* = −0.07, 95% CI: −0.54 to 0.41). In summary, Tai Chi intervention significantly lowers average NRS pain scores in patients with CNLBP, yielding a small-to-medium effect size (*g* = −0.41) (Figure 4).

#### 3.4.3 Pain intensity: peak NRS score

**Figure 5:**
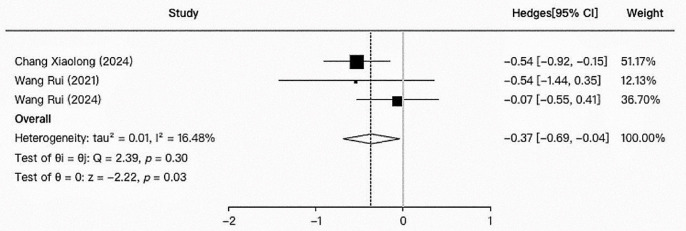
Forest plot of the effect of Tai Chi on peak NRS pain scores in patients with chronic nonspecific low back pain.

Three studies (Chang Xiaolong (21), Wang Rui (22), and Wang Rui (23)) reported data for the “peak” pain intensity dimension measured by the NRS. Heterogeneity testing showed *I*^2^ = 16.48% and *P* = 0.30, indicating low inter-study heterogeneity; thus, a fixed-effects model was utilized for the meta-analysis. The results demonstrated that Tai Chi significantly reduced peak NRS scores compared to the control group (Hedges' *g* = −0.37, 95% CI: −0.69 to −0.04, *P* = 0.03). In summary, Tai Chi intervention significantly decreases peak NRS pain scores in CNLBP patients, with a small-to-medium effect size (*g* = −0.37) (Figure 5).

### 3.5 Functional disability outcomes

#### 3.5.1 Functional disability: RMDQ score

A total of five studies involving 381 patients with chronic non-specific low back pain (CNLBP) were included in the analysis of Roland–Morris Disability Questionnaire (RMDQ) scores. Heterogeneity analysis indicated moderate heterogeneity among studies (*I*^2^ = 57.01%, *Q* = 9.30, *P* = 0.054); therefore, a random-effects model was applied.

**Figure 6:**
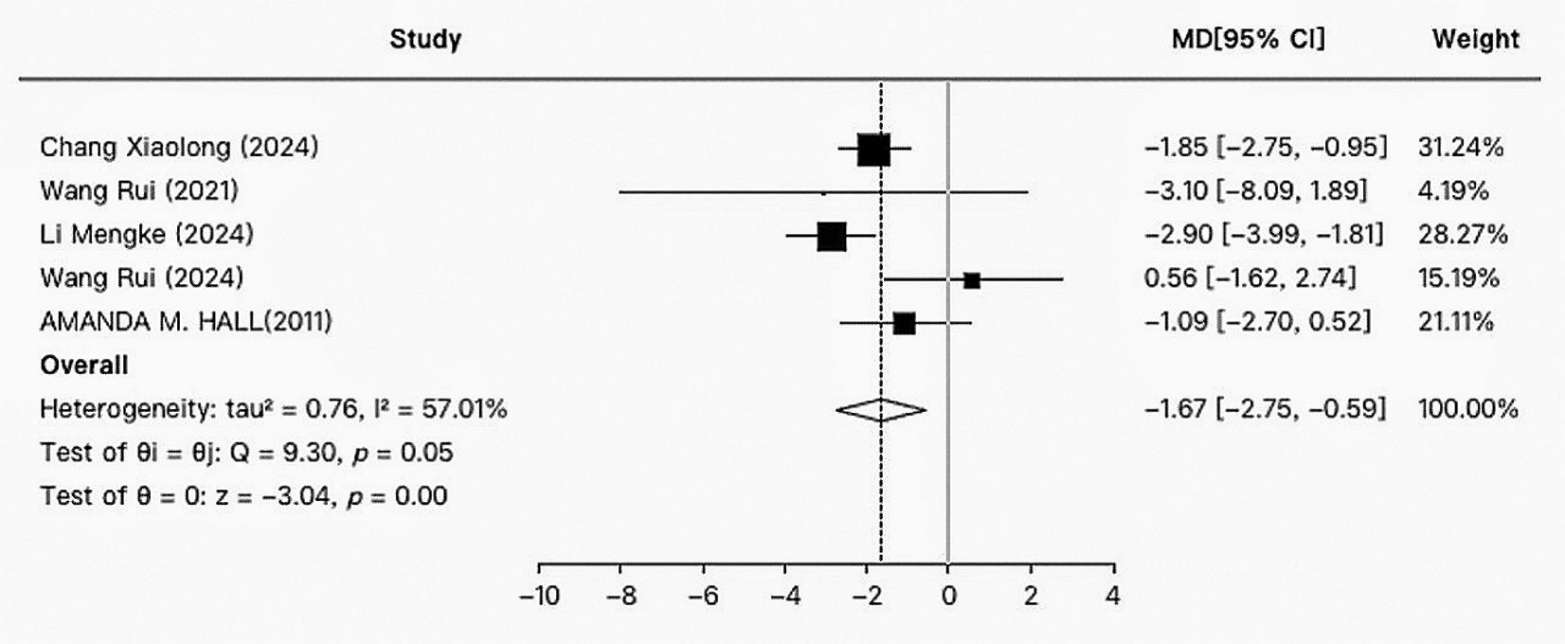
Forest plot for RMDQ disability scores in the Tai Chi and control groups.

The pooled results demonstrated that Tai Chi significantly reduced RMDQ scores compared with the control interventions, with a pooled mean difference of MD = −1.67 (95% CI: −2.75 to −0.59). The difference was statistically significant (*Z* = −3.04, *P* = 0.002), indicating that Tai Chi effectively improved functional disability in patients with CNLBP (Figure 6).

Sensitivity analysis showed that the pooled effect size remained stable after sequential exclusion of individual studies, with MD values ranging from −1.24 to −2.09. The direction and significance of the effect did not change. Publication bias was not formally assessed due to the small number of studies, and thus these analyses are not reported.

#### 3.5.2 Functional disability: ODI score

Three studies involving 129 patients with chronic non-specific low back pain (CNLBP) were included to assess the effect of Tai Chi on Oswestry Disability Index (ODI) scores. High heterogeneity was observed among studies (I^2^ = 72.12%, Q = 7.17, *P* = 0.028); thus, a random-effects model was used.

**Figure 7:**
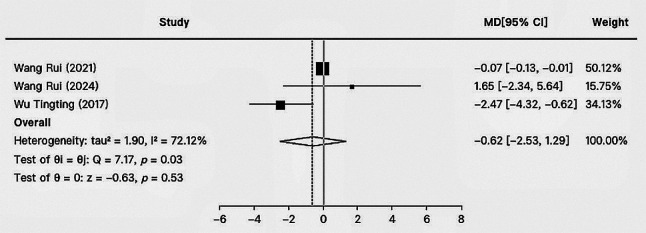
Forest plot of the effect of Tai Chi on ODI disability scores in patients with chronic nonspecific low back pain.

The pooled analysis showed a mean difference of MD = −0.62 (95% CI: −2.53 to 1.29), which was not statistically significant (*Z* = −0.63, *P* = 0.526), indicating that Tai Chi did not significantly improve ODI scores (Figure 7).

Sensitivity analysis revealed that sequential exclusion of individual studies produced pooled effect sizes ranging from MD = −0.07 to −1.085, with no substantial change in effect direction, suggesting good result stability. Publication bias was not formally assessed due to the small number of studies, and thus these analyses are not reported.

#### 3.5.3 Functional disability: combined RMDQ and ODI scores and subgroup analysis

**Figure 8:**
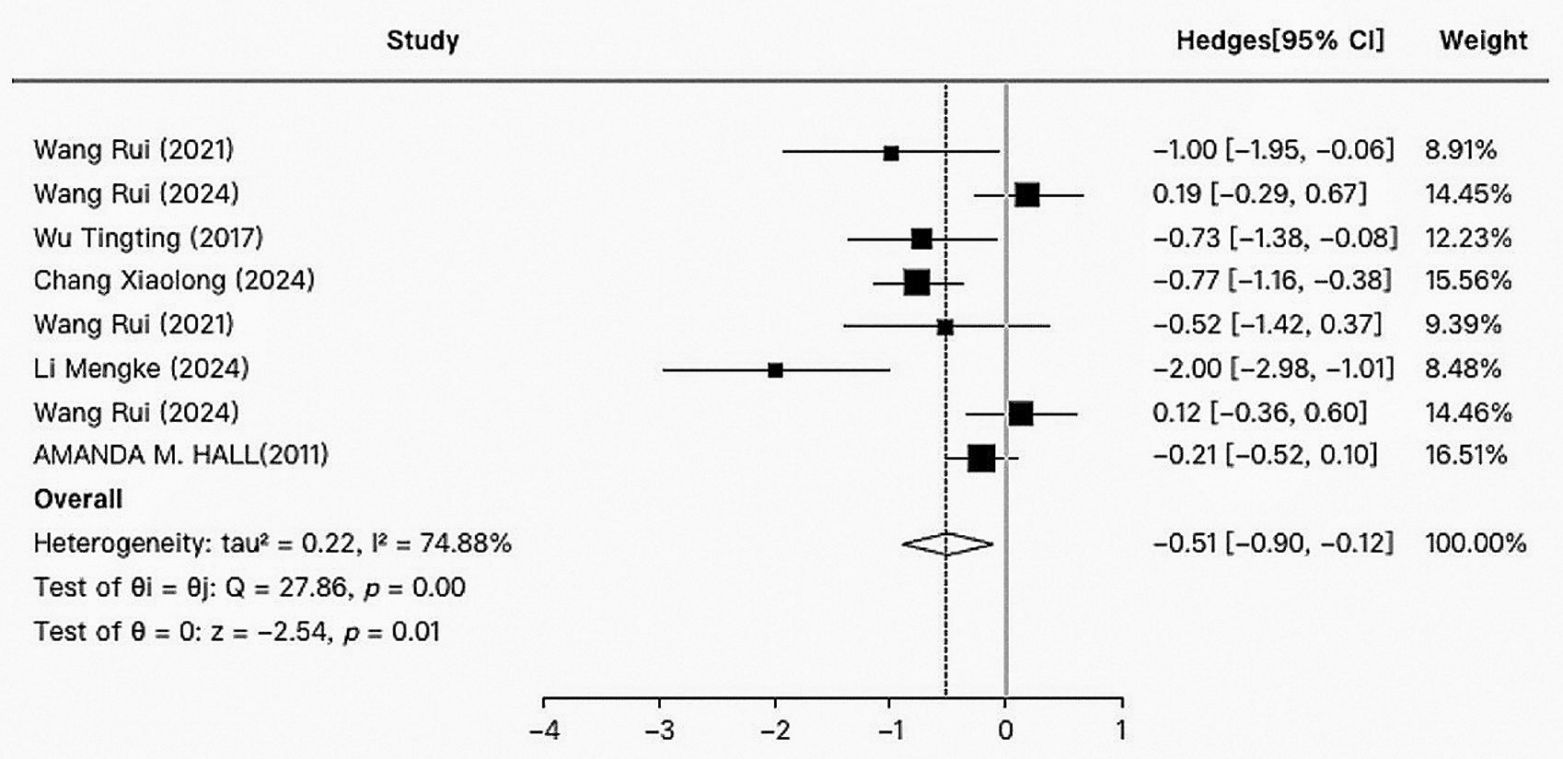
Forest plot of the effects of Tai Chi on RMDQ and ODI scores in patients with chronic non-specific low back pain.

Eight studies involving 510 patients with chronic non-specific low back pain (CNLBP) were included. As studies used different measures of functional disability (RMDQ and ODI), standardized mean differences (SMD) were calculated for the meta-analysis. High heterogeneity was observed among studies (*I*^2^ = 74.88%, *Q* = 27.87, *P* < 0.001); therefore, a random-effects model was applied. The pooled results indicated that Tai Chi significantly improved functional disability, with a combined effect size of SMD = −0.51 (95% CI: −0.90 to −0.12; *Z* = −2.54, *P* = 0.011) (Figure 8).

Sensitivity analysis showed that sequential removal of individual studies resulted in pooled SMDs ranging from −0.35 to −0.62, with the effect direction remaining consistent, indicating good robustness. Publication bias was not formally assessed due to the small number of studies, and thus these analyses are not reported.

Due to high heterogeneity in ODI scores (*I*^2^ = 72.12%) and in the combined functional disability outcomes (RMDQ and ODI, *I*^2^ = 74.88%), subgroup analyses were conducted for functional disability (RMDQ and ODI) across eight studies involving 510 patients (Table 6). Tai Chi showed a favorable effect overall, with standardized mean differences indicating improvement in functional outcomes.

**Table 6:** Subgroup analysis of the effects of Tai Chi on functional disability (RMDQ and ODI scores) in patients with chronic non-specific low back pain.

Outcome	Moderators	Subgroup	No. of Studies (k)	Effect Size Hedges' g [95% CI]	I²
Functional Impairment	Duration (cov1)	Subgroup 1 (≤8 weeks)	2	−1.306 [−2.543, −0.069]	77.36%
		Subgroup 2 (>8 weeks)	6	−0.299 [−0.661, 0.064]	67.21%
	Frequency (cov2)	Subgroup 1 (≤3 times/week)	7	−0.482 [−0.915, −0.049]	77.39%
		Subgroup 2 (>3 times/week)	1	−0.729 [−1.378, −0.080]	0.00%
	Session Length (cov3)	Subgroup 1 (≤45 min)	2	−1.038 [−2.786, 0.709]	91.27%
		Subgroup 2 (>45 min)	6	−0.400 [−0.833, 0.033]	69.53%
	Intervention Type (cov4)	Subgroup 1 (Pure Tai Chi)	7	−0.482 [−0.915, −0.049]	77.39%
		Subgroup 2 (Combined Tai Chi)	1	−0.729 [−1.378, −0.080]	0.00%

Subgroup 1 and Subgroup 2 were classified according to intervention duration (≤8 weeks vs. >8 weeks), training frequency (≤3 times/week vs. >3 times/week), session length (≤45 min vs. >45 min), and intervention type (pure Tai Chi vs. combined Tai Chi intervention). Hedges' g < 0 indicates a favorable effect of Tai Chi on functional impairment.

For intervention duration, the ≤8-week subgroup showed a larger effect (Hedges' *g* = −1.306, 95% CI: −2.543 to −0.069) compared with the >8-week subgroup (Hedges' *g* = −0.299, 95% CI: −0.661 to 0.064), although the between-subgroup difference was not statistically significant. Regarding training frequency, both ≤3 sessions/week (Hedges' *g* = −0.482, 95% CI: −0.915 to −0.049) and >3 sessions/week (Hedges' *g* = −0.729, 95% CI: −1.378 to −0.080) subgroups demonstrated favorable effects, but the high-frequency subgroup included only one study. For session length, neither ≤45 min (Hedges' *g* = −1.038, 95% CI: −2.786 to 0.709) nor >45 min (Hedges' *g* = −0.400, 95% CI: −0.833 to 0.033) reached statistical significance. Both pure Tai Chi (Hedges' *g* = −0.482, 95% CI: −0.915 to −0.049) and combined Tai Chi interventions (Hedges' *g* = −0.729, 95% CI: −1.378 to −0.080) showed improvements, with the combined group based on a single study.

These exploratory subgroup analyses suggest that intervention duration, training frequency, and intervention type may partially influence functional outcomes; however, findings should be interpreted cautiously due to limited study numbers and heterogeneity.

### 3.6 Sleep quality: PSQI score

A total of three studies involving 196 patients with chronic non-specific low back pain (CNLBP) were included in the analysis of sleep quality. Heterogeneity testing indicated moderate heterogeneity among studies (*I*^2^ = 59.75%, *Q* = 4.97, *P* = 0.083); therefore, a random-effects model was used for the meta-analysis.

**Figure 9:**
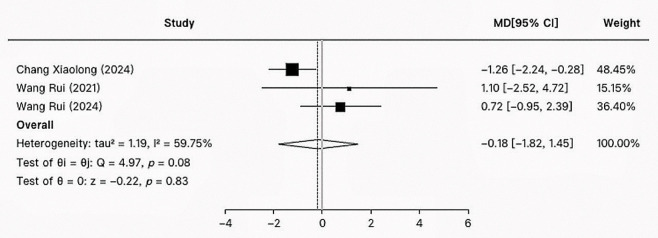
Forest plot comparing PSQI sleep quality scores between Tai Chi and control groups.

The pooled results showed that Tai Chi did not significantly improve Pittsburgh Sleep Quality Index (PSQI) scores, with a pooled mean difference of MD = −0.18 (95% CI: −1.82 to 1.45). The difference was not statistically significant (*Z* = −0.22, *P* = 0.828), suggesting that current evidence is insufficient to demonstrate a significant effect of Tai Chi on sleep quality in patients with CNLBP (Figure 9).

Sensitivity analysis indicated that the pooled effect size ranged from MD = −0.75 to 0.79 after sequential exclusion of individual studies. Neither the direction nor the statistical significance of the effect changed substantially, indicating good stability of the findings. Publication bias was not formally assessed due to the small number of studies, and thus these analyses are not reported.

## 4 Discussion

This study included eight randomized controlled trials involving 526 patients with chronic non-specific low back pain (CNLBP) and systematically evaluated the effects of Tai Chi on pain, functional disability, and sleep quality. The results indicated that Tai Chi significantly reduced pain and improved functional disability. However, no statistically significant improvements were observed in ODI scores or sleep quality.

Although the findings suggest that Tai Chi may contribute to the comprehensive rehabilitation of patients with CNLBP, caution is warranted when interpreting these results due to high heterogeneity in some outcome measures and the limited number of included studies. Overall, the study provides further support for Tai Chi as a safe, low-risk, and sustainable non-pharmacological intervention for the management of CNLBP.

### 4.1 Intervention effects of Tai Chi on pain

The meta-analysis results indicate that Tai Chi can significantly reduce pain in patients with chronic non-specific low back pain (CNLBP). Pooled VAS scores showed that pain intensity in the Tai Chi group was significantly lower than in the control group (MD = −1.40, 95% CI: −2.41 to −0.40, *P* = 0.01). In addition, both the average pain dimension (Hedges' *g* = −0.41) and the worst pain dimension (Hedges' *g* = −0.37) of the NRS reached statistical significance. These findings suggest that Tai Chi has a consistent and positive effect on alleviating subjective pain perception in patients with CNLBP (28).

The observed pain relief may be related to the unique mind-body mechanisms of Tai Chi (29). On one hand, the slow and continuous movements of Tai Chi strengthen the core muscles of the trunk and improve spinal stability, which helps correct abnormal movement patterns and optimizes lumbar load distribution (30). On the other hand, Tai Chi emphasizes controlled breathing and focused attention, which can reduce sympathetic nervous system activity, relieve muscle tension, and alleviate psychological stress. In addition, growing evidence suggests that Tai Chi may modulate central pain processing and increase pain tolerance, thereby contributing to its analgesic effects (31).

It is noteworthy that high heterogeneity was observed in VAS outcomes (*I*^2^ = 95.44%). Subgroup analyses indicated that intervention duration, training frequency, session length, and intervention type may all influence the analgesic effect; however, none of these factors fully explained the overall heterogeneity. Other factors, such as participants' age, disease duration, Tai Chi style, and differences in control interventions, may also contribute. Therefore, although the analgesic effect of Tai Chi is evident, caution is warranted when interpreting the magnitude of the effect (32).

The present findings are generally consistent with previous systematic reviews and meta-analyses reporting beneficial effects of Tai Chi on pain relief in individuals with chronic low back pain. Previous studies have shown that Tai Chi can produce modest but clinically meaningful reductions in pain intensity, supporting its role as a non-pharmacological treatment option. Our findings further strengthen this evidence by focusing specifically on patients with chronic non-specific low back pain and incorporating several recently published randomized controlled trials.

### 4.2 Impact of Tai Chi on functional disability

Functional disability is a major clinical manifestation of chronic non-specific low back pain (CNLBP) and an important factor affecting patients' quality of life and social participation. In this study, RMDQ scores, ODI scores, and the combined functional disability outcome were analyzed separately. The results showed that Tai Chi significantly improved RMDQ scores (MD = −1.67, 95% CI: −2.75 to −0.59, *P* = 0.002), indicating improvements in patients' ability to perform daily activities. However, no statistically significant effect was observed for ODI scores (MD = −0.62, 95% CI: −2.53 to 1.29, *P* = 0.526). The discrepancy between the two measures may be related to differences in their assessment focus. The RMDQ primarily evaluates limitations in daily activities and is more sensitive to mild-to-moderate changes in functional status. In contrast, the ODI places greater emphasis on the overall impact of pain on quality of life and social functioning, and therefore may be less responsive to short-term intervention effects. After combining RMDQ and ODI outcomes using standardized mean differences, the pooled analysis demonstrated that Tai Chi significantly improved overall functional disability (SMD = −0.51, 95% CI: −0.90 to −0.12, *P* = 0.011). These findings suggest that, overall, Tai Chi has a beneficial effect on functional recovery in patients with CNLBP (33).

Subgroup analyses suggested that interventions lasting ≤8 weeks, training frequencies of ≤3 sessions per week, and pure Tai Chi interventions were associated with favorable effects on functional disability. However, these findings should be interpreted with caution because several subgroups included only a small number of studies. In contrast, neither of the session-length subgroups demonstrated statistically significant effects. These findings suggest that intervention duration, training frequency, and intervention type may be important sources of heterogeneity in functional disability outcomes. The beneficial effects of Tai Chi on functional disability may be attributed to several mechanisms. Tai Chi can enhance core stability, improve proprioception, and strengthen motor control. In addition, it may reduce fear of movement and increase patients' confidence in physical activity. Together, these effects may facilitate the recovery of normal functional abilities and improve daily physical performance in patients with CNLBP (34).

Similar findings have been reported in previous reviews, which demonstrated that Tai Chi may improve physical function and reduce disability in patients with chronic low back pain. However, unlike some previous studies, the present analysis did not identify a significant improvement in ODI scores. This discrepancy may be related to differences in measurement characteristics between the RMDQ and ODI instruments.

### 4.3 Impact of Tai Chi on sleep quality

Sleep disturbance is a common comorbidity in patients with chronic non-specific low back pain (CNLBP), and a bidirectional relationship exists between pain and sleep. However, the present study found that Tai Chi did not significantly improve PSQI scores (MD = −0.18, 95% CI: −1.82 to 1.45, *P* = 0.828). This finding is not entirely consistent with some previous studies that have reported beneficial effects of Tai Chi on sleep quality. Several factors may explain this discrepancy. First, only three studies reporting PSQI outcomes were included, resulting in limited statistical power. Second, sleep quality was not the primary outcome in any of the included studies, and the intervention protocols were mainly designed to target pain relief and functional improvement. Third, differences in baseline sleep disturbance among participants may have attenuated the observed intervention effects. In addition, the Pittsburgh Sleep Quality Index (PSQI) is a subjective assessment tool and may be influenced by individual perceptions, cognitive factors, and psychological status (35).

Although the pooled effect estimate favored Tai Chi, no statistically significant improvement in PSQI scores was observed. Therefore, the current evidence is insufficient to draw definitive conclusions regarding the effects of Tai Chi on sleep quality in patients with CNLBP. The lack of significance may be attributable to the limited number of included studies, small sample sizes, and substantial variability in baseline sleep characteristics among participants.

Given the limited evidence available, further high-quality randomized controlled trials specifically designed to evaluate sleep outcomes are needed. Future studies should incorporate both subjective and objective sleep assessments to better clarify the potential role of Tai Chi in improving sleep quality among patients with CNLBP.

Previous studies in older adults and patients with chronic diseases have suggested that Tai Chi may improve sleep quality. However, the current meta-analysis did not demonstrate a significant effect on PSQI scores in patients with CNLBP. This discrepancy may be attributable to the limited number of available studies and the fact that sleep quality was not the primary outcome in most included trials.

### 4.4 Heterogeneity analysis

Some outcomes in this study exhibited high heterogeneity, particularly VAS scores (*I*^2^ = 95.44%), ODI scores (*I*^2^ = 72.12%), and the combined functional disability outcome (*I*^2^ = 74.88%). Subgroup analyses suggested that intervention duration, training frequency, and intervention type may partially contribute to this heterogeneity, but they did not fully explain the observed variability.

Potential sources of heterogeneity include differences in Tai Chi styles across studies, intervention durations ranging from 4 to 12 weeks, training frequencies of 2–5 sessions per week, session lengths of 30–65 min, and diverse control interventions, such as health education, usual care, core stability training, acupuncture, or sham Tai Chi. In addition, variations in participant age, disease duration, and baseline pain levels may also influence intervention effects.

Nevertheless, Sensitivity analyses indicated that the main findings were robust. Due to the small number of included studies, publication bias could not be formally assessed (36).

## 5 Limitations and future perspectives

### 5.1 Limitations

This study has several limitations. First, the number of included studies was relatively small, and some outcomes, such as ODI and PSQI, were reported in only three studies, which may limit statistical power and affect result stability. Second, some included studies employed combined interventions, such as Tai Chi with acupuncture or medication, making it difficult to isolate the independent effect of Tai Chi and potentially influencing effect size estimates. Third, variations in Tai Chi style, intervention duration, training frequency, and session length across studies contributed to high heterogeneity in some outcomes. Although subgroup analyses were conducted, they could not fully explain the sources of heterogeneity. Fourth, most studies reported only short-term intervention effects, with limited long-term follow-up data, making it difficult to evaluate the sustained efficacy of Tai Chi for CNLBP. Finally, several included studies were theses or dissertations, which may have limitations in reporting quality and methodological rigor.

### 5.2 Future perspectives

Future research should focus on large-scale, multicenter, high-quality randomized controlled trials with standardized Tai Chi intervention protocols and clearly defined dosage parameters, including optimal intervention duration, training frequency, and session length. Efforts should be made to minimize combined interventions in order to clarify the independent effects of Tai Chi. Additionally, long-term follow-up studies are warranted, and outcome measures should be expanded to include sleep quality, psychological health, and quality of life. Integrating imaging, biomechanical, and neurophysiological assessments may further elucidate the mechanisms by which Tai Chi improves CNLBP, providing stronger evidence to support its clinical application.

## 6 Conclusion

The findings of this systematic review and meta-analysis indicate that Tai Chi can reduce pain intensity and improve functional disability in patients with chronic non-specific low back pain (CNLBP). Significant benefits were observed for VAS scores, RMDQ scores, and the pooled functional disability outcome. However, no statistically significant improvements were found for ODI scores or PSQI scores. Subgroup analyses suggested that intervention duration, training frequency, and intervention type may influence treatment effects and partially explain the heterogeneity observed across studies.

Given the limited number of included studies, the use of combined interventions in some trials, and the substantial heterogeneity observed in several outcomes, the current evidence should be interpreted with caution. Overall, Tai Chi appears to be a safe and feasible non-pharmacological rehabilitation strategy for patients with CNLBP. Nevertheless, further high-quality randomized controlled trials are needed to determine the optimal intervention protocol and to evaluate its long-term effectiveness.

## Data Availability

The original contributions presented in the study are included in the article/Supplementary Material, further inquiries can be directed to the corresponding author/s.
